# Interface Engineering through Atomic Layer Deposition towards Highly Improved Performance of Dye-Sensitized Solar Cells

**DOI:** 10.1038/srep12765

**Published:** 2015-08-04

**Authors:** Hao Lu, Wei Tian, Jun Guo, Liang Li

**Affiliations:** 1 College of Physics, Optoelectronics and Energy, Jiangsu Key Laboratory of Thin Films, Soochow University, China; 2 Analysis and Testing Center of Soochow University, Soochow University, China

## Abstract

A composite photoanode comprising ultralong ZnO nanobelts and TiO_2_ nanoparticles was prepared and its performance in dye-sensitized solar cells (DSSCs) was optimized and compared to the photoanode consisting of conventional TiO_2_ nanoparticles. The ultralong ZnO nanobelts were synthesized in high yield by a facile solution approach at 90 ^o^C followed by annealing at 500 ^o^C. The effect of the ratio of ZnO nanobelts to TiO_2_ nanoparticles on the light scattering, specific surface area, and interface recombination were investigated. An optimum amount of ZnO nanobelts enhanced the photon-conversion efficiency by 61.4% compared to that of the conventional TiO_2_ nanoparticles. To further reduce the recombination rate and increase the carrier lifetime, Atomic Layer Deposition (ALD) technique was utilized to coat a continuous TiO_2_ film surrounding the ZnO nanobelts and TiO_2_ nanoparticles, functioning as a barrier-free access of all electrons to conductive electrodes. This ALD treatment improved the interface contact within the whole photoanode system, finally leading to significant enhancement (137%) in the conversion efficiency of DSSCs.

Dye-sensitized solar cells (DSSCs) usually consist of a photoanode with a transparent TiO_2_ nanoparticle film sensitized with visible light harvesting dye, a redox electrolyte, and a platinum (Pt) counter electrode^1^,^2^,^3^. Efficiency reaching to 13% has already been achieved in laboratory through the molecular engineering of porphyrin sensitizers^4^, whereas the overall performance is still low owing to poor utilization of solar light and recombination losses occurring in the random network of TiO_2_ nanoparticle films. Although nanoparticles provide large surface area for high amount of dye loading, the existence of numerous boundaries (Fig. 1(a)) in the nanoparticle films increases charge recombination occurred at the interfaces of TiO_2_ nanoparticles/dye/electrolyte. It is estimated that an electron visits about 10^6^ nanoparticles in a 10 μm thick film before reaching the FTO substrate^5^. Employing one- or two- dimensional (1D or 2D) nanostructures as photoanodes, including nanowires, nanorods and nanosheets, is an effective route to resolve the above problem because they provide direct electron transport pathways from the site occurring electron injection to the collector electrode and suppress the interfacial charge recombination^6^,^7^,^8^,^9^. However, the main disadvantage of 1D or 2D nanostructures is their lower surface area (when compared with nanoparticle films) for insufficient adsorption of light-harvesting molecules, leading to relatively low conversion efficiency.

Recent studies have shown that ZnO is a promising alternative photoanode material, which is attributed to the following facts: i) both ZnO and TiO_2_ have similar physical properties and almost the same band gap energies, ii) the electron mobility of ZnO is higher than that of TiO_2_, resulting in lower charge recombination, iii) various ZnO nanostructures could be realized due to ease of crystallization originating from their anisotropic growth^10^,^11^,^12^. However, till now, the conversion efficiencies obtained for ZnO-based DSSCs are still relatively low compared with TiO_2_-based systems. This may be explained by the slow electron-injection kinetics from dye to ZnO, and the dissolution of ZnO in acidic dye leading to the formation of excessive Zn^2+^/dye agglomerates^13^,^14^. Therefore, it is difficult to further increase the conversion efficiency of ZnO-based DSSCs when using ZnO-only as photoanode materials. Taking advantage of high charge transfer efficiency of 1D ZnO nanostructures and large surface area of TiO_2_ nanoparticles, hybrid photoanodes consisting of their composites have been investigated and an enhancement of conversion efficiency was clearly demonstrated, while only 15–26.9% improvement was observed compared to conventional TiO_2_ nanoparticle films^15^,^16^,^17^. The poor enhancement could arise from these factors: i) the amount of dye loading is decreased with increasing the relative ratio of 1D ZnO nanostructures to TiO_2_ nanoparticles in films, ii) as shown in Fig. 1(b), the cylindrical 1D ZnO nanostructures and the spherical TiO_2_ nanoparticles form a poor interface (point-to-point) contact, which is not favorable for charge transfer, iii) the instability of ZnO in dye further prevents the charge transport.

In this article, we present a solution reaction process for high-yield synthesis of superlong ZnO nanobelts in a water bath at 90 ^o^C followed by annealing at 500 ^o^C. Then, the composites of the ZnO nanobelts and P25 TiO_2_ nanoparticles were used as the photoanodes of DSSCs. Our results showed a large enhancement of 61.4% in conversion efficiency over the conventional TiO_2_-based DSSCs. Furthermore, such composites were coated with a TiO_2_ thin film by ALD technique to improve interface connection in the system, and a significantly enhanced efficiency of 137% was achieved. We also systematically investigated the effect of the relative nanobelts/nanoparticles ratios on the performance of DSSCs, and a proposed mechanism was discussed in detail *via* current-voltage curves, incident photon-to-current conversion efficiency, reflectance and transmittance spectra, and electrochemical impendence spectroscopy measurements.

## Results and Discussion

### Synthesis and characterization of ultralong ZnO nanobelts

Figure 2 (a,b) demonstrate the representative SEM images of as-synthesized ZnO nanobelts. It can be seen from a low–magnification image that the nanobelts are very long and the length is estimated to be over tens of micrometers. High-magnification image indicates that these nanobelts are highly flexible and can be bent in an arbitrary angle. This flexible characteristics is important for getting a homogeneous composite of 1D nanostructures and other dimensional materials. Figure 2(c) is a digital photo of the sample, showing the solution was filled with white flocculent ZnO nanobelts after a simple low-temperature solution reaction process. In the XRD pattern (Fig. 2(d)), the diffraction peaks of as-annealed samples can be indexed to (100), (002), (101), and other planes of hexagonal wurtzite ZnO, according to the Joint Committee on Powder Diffraction standards (JCPDS 79-2205)^18^. Transmission electron microscopy (TEM) and high-resolution TEM (HRTEM) images of nanobelts are shown in Fig. 2(e,f), respectively. TEM image shows that two nanobelts overlap, and the belt feature makes the one underneath visible clearly. HRTEM image represents that ZnO nanobelts are single-crystalline with a [100] growth direction.

### ZnO nanobelt/TiO2 nanoparticle composite photoanodes

**Figure 1:**
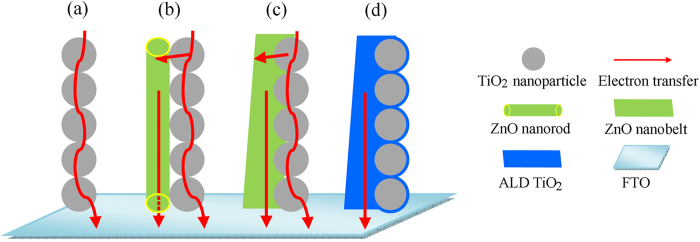
Schematic of electron transport in (**a**) the conventional TiO_2_ nanoparticle, (**b**) ZnO nanowire/TiO_2_ nanoparticle composite, (**c**) ZnO nanobelt/TiO_2_ nanoparticle composite, and (**d**) ZnO nanobelt/TiO_2_ nanoparticle composite coated with TiO_2_ film by ALD.

**Figure 2:**
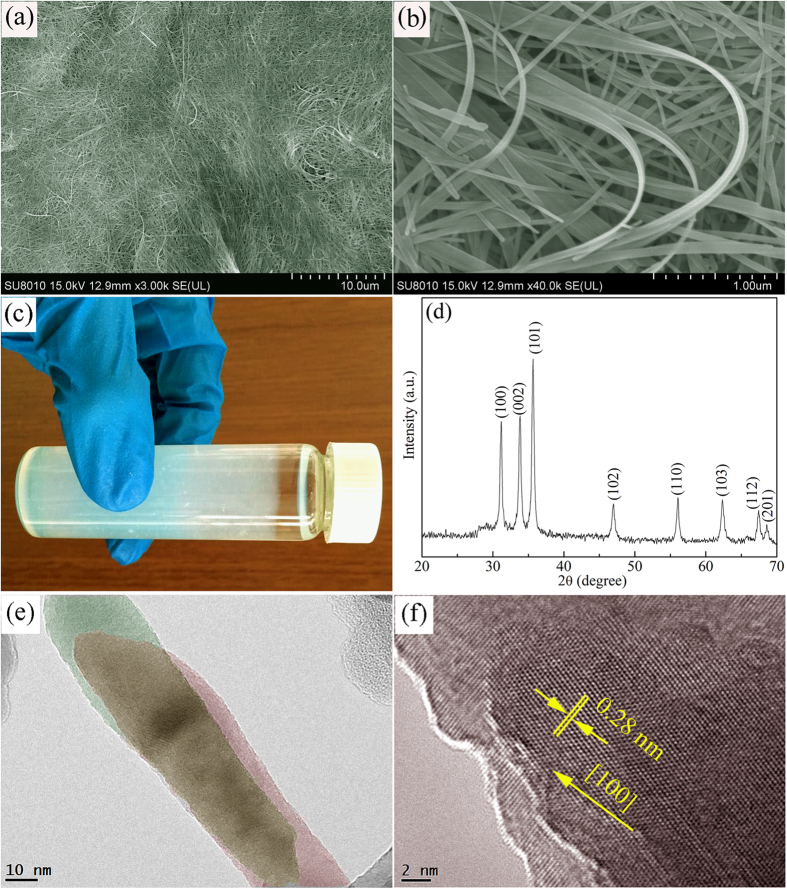
(**a**) SEM image of ZnO nanobelts showing the ultralong length. (**b**) High-magnification image of nanobelts demonstrating the flexible characteristic. (**c**) A photograph of the reagent bottle filled with ZnO nanobelts solution. (**d**) XRD patterns of as-synthesized ZnO nanobelts. (**e**) TEM image of two overlapped nanobelts and (**f**) corresponding HRTEM image indicating the nanobelts are single-crystalline and the thickness is thin.

**Figure 3:**
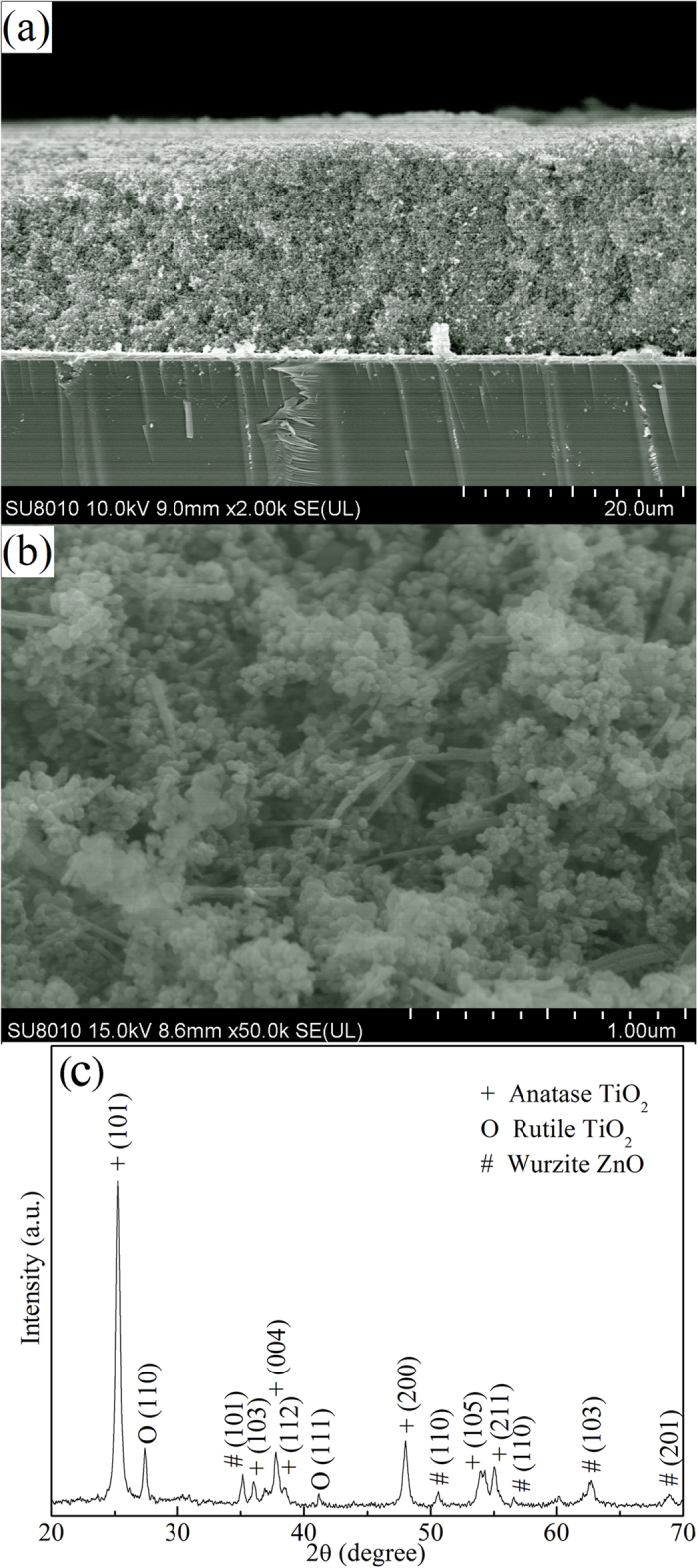
(**a**) Cross-sectional SEM image, (**b**) High-magnified SEM image and (**c**) XRD pattern of ZnO nanobelt/P25 TiO_2_ nanoparticle composite film on a substrate.

Figure 3(a) shows the typical cross-sectional SEM image of the composite of ZnO nanobelts/TiO_2_ nanoparticles (described as ZnO0.5), from which it is seen that the film thickness is about 30 μm. The thickness of samples with different ZnO weight rate could be found in Figure S1. As seen, all these samples have nearly the same thickness. Owing to the ultralong length, nanobelts can facilitate the direct transport of carriers from the composite to the FTO conductive substrate. Compared with 1D ZnO nanowires, ZnO nanobelts can provide more contact area for 0D nanoparticles (Fig. 1(c)) and thus more efficient carrier transfer. Figure 3(b) shows high-magnified image of the composite, indicating homogeneous distribution of ZnO nanobelts and TiO_2_ nanoparticles. Figure 3(c) shows a XRD pattern of the composite printed on a FTO substrate after annealing in 500 ^o^C. As indicated in the XRD pattern, all diffraction peaks of the composite are well indexed to a mixture of hexagonal wurtzite ZnO, anatase TiO_2_ (JCPDS 21-1272), and rutile TiO_2_ (JCPDS 21-1276)^19^.

**Figure 4:**
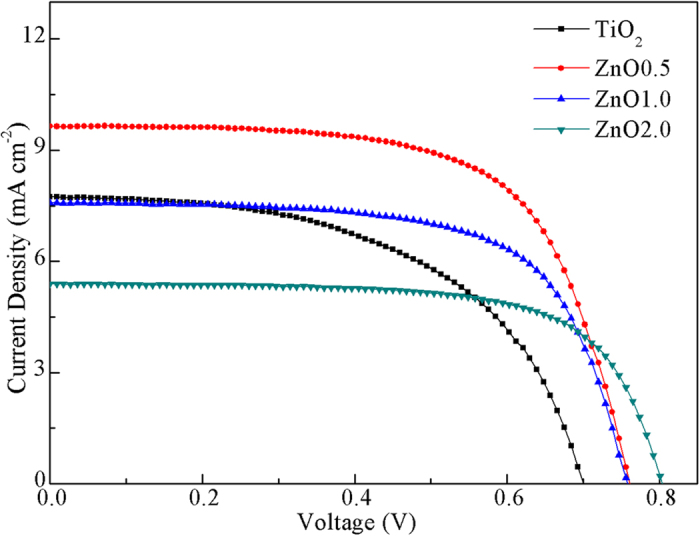
*J*–*V* curves of DSSCs based on pristine TiO_2_ nanoparticles and ZnO nanobelt/TiO_2_ nanoparticle composites (ZnO0.5, ZnO1.0, and ZnO2.0) photoanodes.

Figure 4 shows the measured current density–bias voltage (*J*–*V*) curves of DSSCs using the pristine TiO_2_ nanoparticles and ZnO nanobelt/TiO_2_ nanoparticle composites as photoanodes, revealing the effect of ZnO amounts (ZnO0.5: 5.0 wt% of ZnO; ZnO1.0: 10 wt% of ZnO; ZnO2.0: 20 wt% of ZnO) on the photoelectrochemical performances. The detailed parameters, including open–circuit voltage (*V*_oc_), short–circuit current density (*J*_sc_), fill factor (*FF*) and energy conversion efficiency (*η*) are listed in Table 1. A *η* of 2.90% was achieved for the pristine TiO_2_ nanoparticle film, with a *J*_sc_ of 7.74 mA/cm^2^, a *V*_oc_ of 0.70 V and a *FF* of 53.6. With increasing amount of ZnO nanobelts, both *V*_oc_ and *FF* increased, but *J*_sc_ and *η* values increased first and then decreased. When the amount of ZnO nanobelts is 5.0%, the *J*_sc_ and *η* have the largest values of 9.67 mA/cm^2^ and 4.68%, respectively. Obviously, a proper amount of ZnO nanobelts largely enhance the conversion efficiency by 61.4% compared to that of conventional TiO_2_ nanoparticles.

The *V*_oc_ value of a DSSC is proportional to the difference between Fermi level for electrons in the photoanode material and the redox potential of I^−^/I_3_^−^. This means that the *V*_oc_ for a given photoelectrode–electrolyte system is a constant. However, experimental results have indicated that the *V*_oc_ usually depends on the recombination rate^20^,^21^. The higher *V*_oc_ with increased amount of ZnO nanobelts can be explained as a consequence of reduced interfacial recombination (confirmed by the following EIS results) due to the excellent electron transfer in the interconnected network of nanobelts, which results in an increase of electron density in the composite and thus the negative shift of Fermi level. Enhancement of *V*_oc_ was also observed in TiO_2_ nanotube films by Ohsaki *et al.*^22^. The *FF* is determined by the internal resistance of cells. The internal resistances of these composite cells become lower with increasing the amount of ZnO nanobelts since more electrons can transport rapidly through nanobelt networks. The key to high *J*_sc_ lies in a large amount of dye adsorption, sufficient light harvesting, and fast charge transport. The amount of adsorbed N719 dyes was evaluated by measuring the eluted dye molecules from photoanodes with UV–vis absorption spectroscopy (Figure S2). The calculated dye concentrations are 80.5, 81.7, 101.5, and 165.9 nmol/cm^2^ for TiO_2_, ZnO0.5, ZnO1.0 and ZnO2.0, respectively (Table 2). Obviously, all composites can absorb more dye molecules than pristine TiO_2_ nanoparticles. The amount of dye loading increases when ZnO nanobelts content are increased in composites, implying that ZnO nanobelts have outstanding absorption capacity although it is generally believed that 1D nanostructures have lower specific surface area than nanoparticles (Figure S3). In particular, ZnO2.0 sample shows 1.1 times higher dye loading than pristine TiO_2_ nanoparticles.

**Figure 5:**
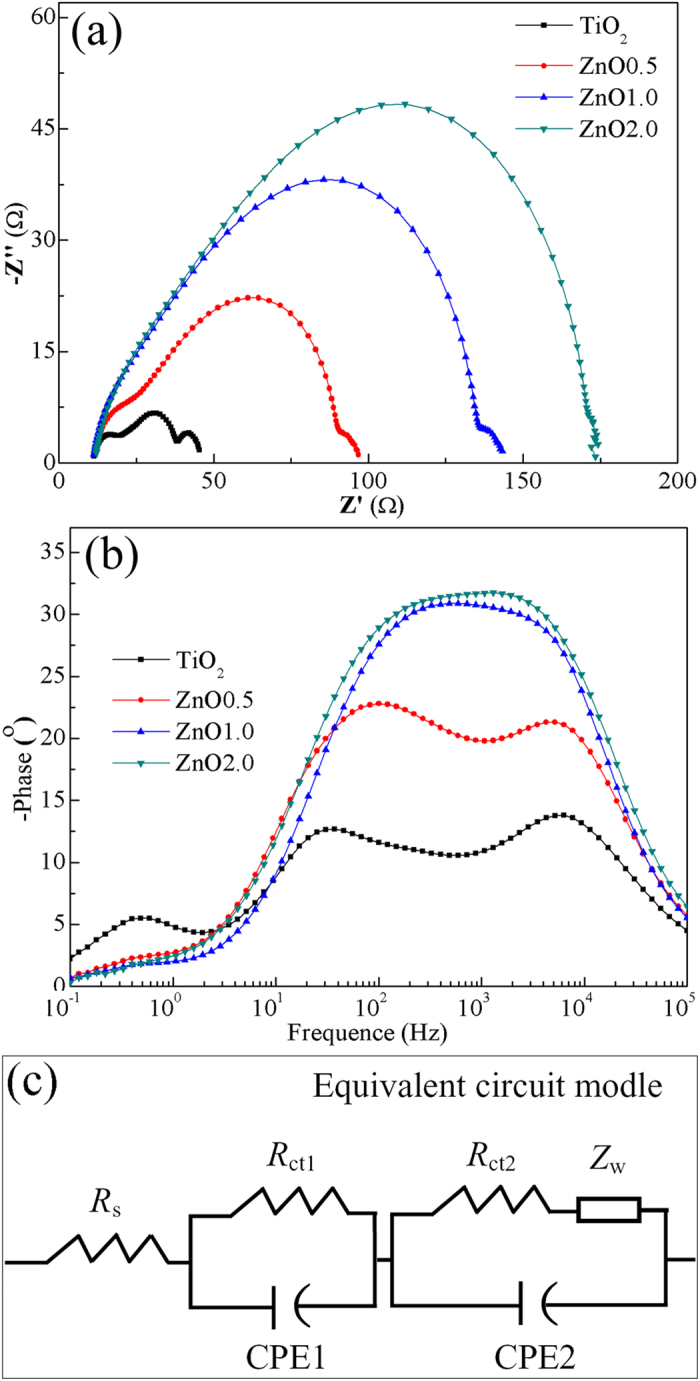
(**a**) Nyquist and (**b**) Bode phase plots of DSSCs with the pristine TiO_2_ nanoparticles and ZnO0.5, ZnO1.0, and ZnO2.0 photoanodes obtained at an open-circuit voltage in dark. (**c**) The equivalent circuit for fitting measured EIS plots.

In order to explore the kinetics of photoelectrochemical processes, the electrochemical impedance spectroscopy (EIS) was performed under dark and open–circuit voltage. The mechanism is based on the widely used diffusion-recombination model^23^. In the dark, the electrons are injected from the FTO substrate into the TiO_2_ conduction band upon forward bias and then the TiO_2_ film is charged by electron accumulation. Finally, some of these injected electrons are lost by the charge recombination with I_3_^−^ ions in the electrolyte. This means that the higher the recombination resistance, the smaller the recombination probability. Figure 5(a) demonstrates that the Nyquist plots have three semicircles in the frequency range of 0.1 Hz–100 kHz. The first semicircle in the high frequency range represents the resistance at the counter electrode (*R*_Pt_) for the reduction reaction of I_3_^−^ ions in the electrolyte using Pt as the counter electrode. The second semicircle in the intermediate frequency range corresponds to the charge transfer resistances at the ZnO (TiO_2_)/dye/electrolyte interface (*R*_ct_). The third semicircle in the low frequency range represents the Warburg diffusion (*Z*_w_) process of the I^−^/I_3_^−^ in the electrolyte. These parameters summarized in Table 2^24^,^25^,^26^. Compared to the cells with pristine TiO_2_ nanoparticles, the *R*_ct_ values are much higher in the case of DSSCs with the network of ZnO nanobelts and increase with the nanobelts amount, indicating a relatively faster electron transfer process at the interface and decreased recombination chances. These results are in consistency with the higher values of *V*_oc_ and *FF* of the DSSCs with composite photoanodes. The electron lifetimes (*τ*_e_) are determined from the Bode-phase plots, as shown in Fig. 5(b), using the following equation^27^,^28^,^29^.


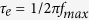


where *f*_max_ is the maximum peak frequency in the middle-frequency range. The measurements revealed that the electron lifetime *τ*_e_ in composite photoanodes was decreased gradually from ZnO0.5 to ZnO2.0. It is noticed that EIS parameters were achieved by fitting using an equivalent circuit model (Fig. 5(c)). Clearly, EIS analysis suggest that as compared to pristine TiO_2_ nanoparticles, the composite nanostructures have lower interface recombination rate and thus increased *V*_oc_ and *FF*, but electron lifetimes are reduced and thus decreased *J*_sc_. From the above results, dye loading, reflectance and electron lifetimes have competitive effects on the *J*_sc_, which first increases and then decreases with the ZnO nanobelt content. The decrease of electron lifetime is attributed to two possible reasons: i) 1D ZnO nanobelts can provide direct charger transport pathways to reduce chances that electrons are reacted with I_3_^–^ in the composites, but extra boundaries between nanobelts and nanoparticles are not beneficial to electron transport and collection, ii) with increasing the nanobelts amount, more nanobelts would not be surrounded by TiO_2_ nanoparticles but are directly exposed to the dye and electrolyte. It is well known that the instability of ZnO in dye and electrolyte results in the slow electron-injection kinetics from dye to ZnO.

**Figure 6:**
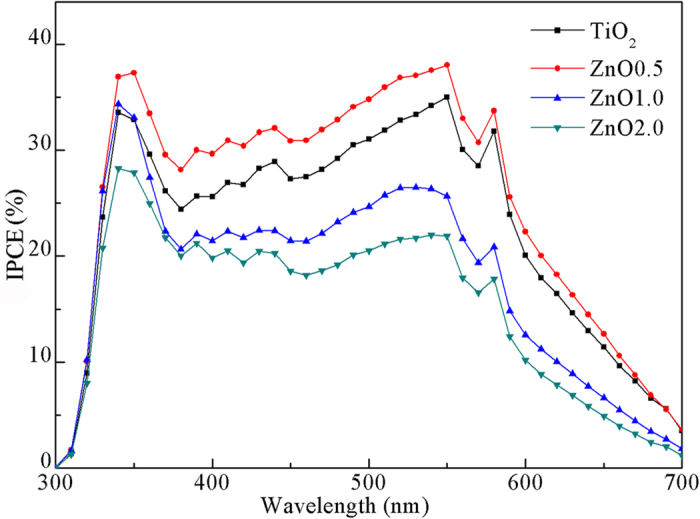
IPCE spectra of DSSCs with the pristine TiO_2_ nanoparticles and ZnO0.5, ZnO1.0, and ZnO2.0 photoanodes.

The incident photon-to-current conversion efficiency (IPCE) spectra of various photoanodes provide further evidence for the electron transport properties in films, as shown in Fig. 6. Generally, the IPCE is defined as the ratio of the number of collected electrons produced by an incident photon at a given wavelength divided by the number of incident photons and can be expressed as the following equation^30^:


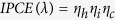


where *η*_h_ is the light harvesting efficiency that is related with the dye loading, *η*_i_ is the injection efficiency of photoexcited electrons from dye to the conduction band of semiconductors, and *η*_c_ is the electron collection efficiency that is related with the electron transport in the system. The IPCE of ZnO0.5 composite is higher than that of the pristine TiO_2_ nanoparticle in the visible light region, while the IPCEs of the ZnO1.0 and ZnO2.0 composites are lower, which has the same trend as the *J*_sc_. When the ZnO nanobelts content is increased, although the values of *η*_h_ gradually increase due to increased dye loading, the IPCEs of the composites first increase and then decrease, indicating either the *η*_i_ or the *η*_c_ have negative effects on IPCE. This is consistent with the above assumed reasons for the decrease of electron lifetimes.

### Interface optimization in ZnO nanobelt/TiO2 nanoparticle system

ALD is a nanoscale deposition technique owing to its ability to fabricate films at a controllable sub-nanometer (usually sub-angstrom) rate and to coat structures that are highly porous and/or tortuous. A variety of applications, including lithium-ion batteries, dye-sensitized solar cells and field-effect transistors, *etc*, have emerged using deposited materials by ALD^31^,^32^,^33^,^34^,^35^. Particularly, interfacial engineering by ALD has led to exciting understanding of interface and surface for solar cells^36^,^37^. Here, we utilized the ALD to modify interfaces in the ZnO nanobelt/TiO_2_ nanoparticle composites for further improving the lifetime of carriers and found a fascinating efficiency enhancement by 137% compared to conventional TiO_2_ nanoparticles.

**Figure 7:**
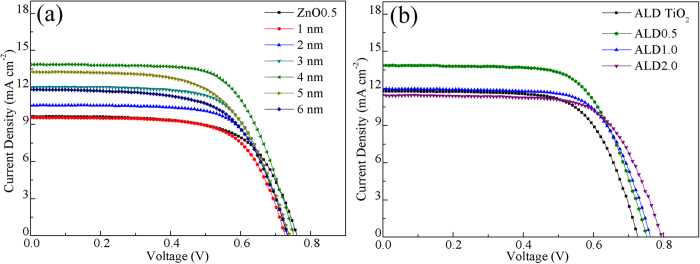
(**a**) *J*–*V* curves of DSSCs based on ZnO0.5 photoanodes coated by TiO_2_ film with the thickness ranging from 1 to 6 nm using ALD. (**b**) *J*–*V* curves of DSSCs based on ALD TiO_2_ nanoparticles and composites (ALDO0.5, ALD1.0, and ALD2.0) photoanodes with coated 4 nm TiO_2_ film.

**Figure 8:**
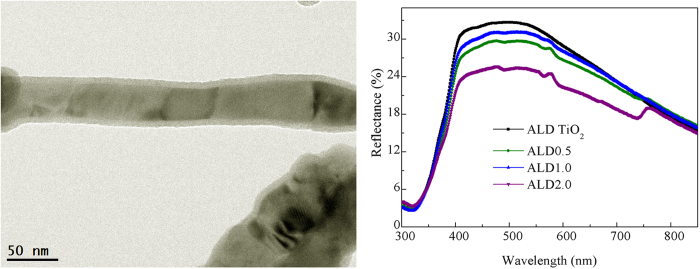
(**a**) HRTEM image showing uniform coating of TiO_2_ outside ZnO nanobelts, with thickness of about 4 nm. (**b**) Diffused reflectance spectra of ALD TiO_2_ nanoparticles, ALDO0.5, ALD1.0, and ALD2.0 composites films.

Figure 7(a) shows the *J*–*V* curves of DSSCs with a ZnO0.5 photoanode coated by TiO_2_ film (the thickness from 1 to 6 nm) using ALD. It is clear that the *η* of DSSCs first increases and then decreases with the increase of TiO_2_ thickness, and the highest *η* value is obtained at 4 nm. Thereafter, we use 4 nm as the TiO_2_ thickness to study the effect of ALD coating on the performance of DSSCs. Figure 7(b) represents the *J*–*V* curves of DSSCs based on ALD-coated ZnO nanobelt/TiO_2_ nanoparticle composites (ALD0.5, ALD1.0 and ALD2.0 correspond to ZnO0.5, ZnO1.0 and ZnO2.0 coated with TiO_2_ film by ALD, respectively). The values of *V*_oc_, *J*_sc_, *FF* and *η* are summarized in Table 3. Compared with the results of cells without ALD coating (Table 1), the *J*_sc_ values of ALD cells are increased largely and the magnitude of enhancement becomes larger with the amount of ZnO nanobelts, leading to similar efficiencies for all composites. Figure 8(a) shows HRTEM image of ZnO nanobelts with ALD TiO_2_ film. The TiO_2_ layer can be easily distinguished on the surface of ZnO nanobelts, with thickness of about 4 nm.

To explore the origin of *J*_sc_ change, we investigated the dye loading and light scattering of ALD composites using UV-vis absorption (Figure S4) and reflectance spectra (Fig. 8(b), respectively. It is found that the absorbed dye amounts (Table 4) of these ALD composites do not change obviously in comparison with those of corresponding cells without TiO_2_ coating (Table 1), which is in well agreement with the BET results. BET surface areas of ZnO0.5 and ALD0.5 photoanodes are very close, with 42.39 and 41.38 m^3^/g, respectively. From the diffused reflectance spectra, ALD composite films have similar reflectance curves with those of films without ALD coating, while the absolute reflectance is slightly higher in the long wavelength range of about 600–800 nm. The possible reason is that a thin layer of ALD film makes all the nanoparticles and nanobelts become a closely interconnected system and thus the effective light scattering sizes are larger than those of individual nanoparticles or nanobelts. This enhancement could significantly extend the traveling distance of incident light within the photoelectrode film and increase the probability of photon harvesting by dye molecules, resulting in a relatively higher *J*_sc_.

**Figure 9:**
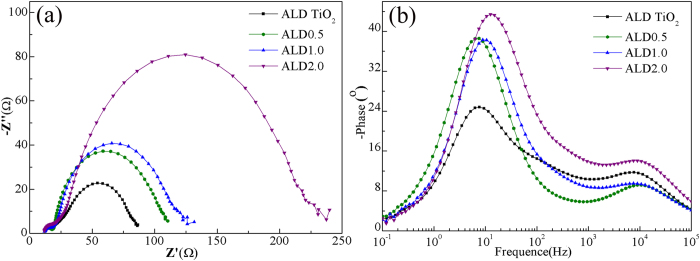
(**a**) Nyquist and (**b**) Bode phase plots of the DSSCs with the ALD TiO_2_ nanoparticles, ALDO0.5, ALD1.0, and ALD2.0 composites photoanodes obtained at an open-circuit voltage in dark.

**Figure 10:**
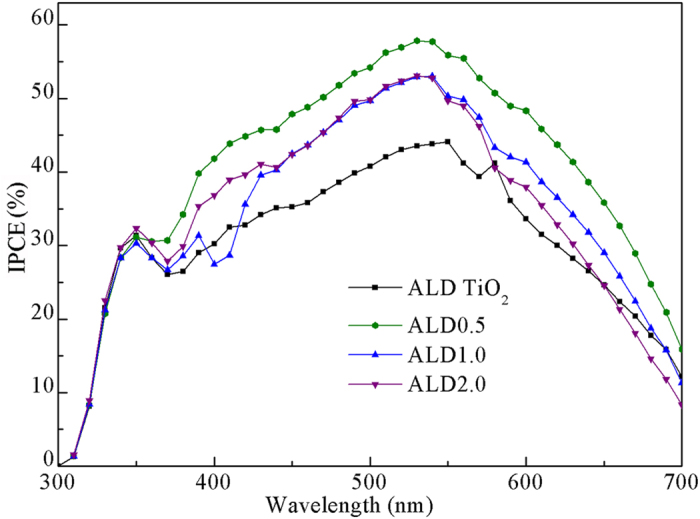
IPCE spectra of DSSCs with the ALD TiO_2_ nanoparticles, ALDO0.5, ALD1.0, and ALD2.0 composites photoanodes.

The EIS analysis of DSSCs with ALD photoelectrodes was carried out under dark and open-circuit voltage. The fitted data of the *R*_s_, *R*_Pt_ and *R*_ct_ obtained from the Nyquist plots (Fig. 9(a) at different nanobelt content are shown in Table 4. We see that the *R*_ct_ values are increased largely compared with those of photoelectrodes without ALD coating. The higher *R*_ct_ values indicate that the charge recombination between the injected electrons and electrons acceptors (I_3_^−^) from the redox electrolyte at the composites/dye/electrolyte interfaces is remarkably retarded. The electron lifetimes *τ*_e_ calculated from the middle frequency peak in Bode plots (Fig. 9(b) are listed in Table 4, showing that: i) ALD cells have significantly increased lifetimes than those of corresponding cells without coating, ii) the electron lifetime in the ALD0.5 is higher than that in pristine TiO_2_ and other ALD composites (ALD1.0 and ALD2.0), iii) although the electron lifetime still decreases with increasing the amount of ZnO nanobelts, their difference of lifetimes becomes no obvious. Combining with the analysis of electron lifetimes in DSSCs without ALD coating, we believe that a continuous TiO_2_ film surrounding all the TiO_2_ nanoparticles and ZnO nanobelts can function as a barrier-free access to all electrons in photoelectrodes, as shown in Fig. 1(d). This ALD film not only protects ZnO from etching in dye solution but also allows ZnO nanobelts to maintain the interior capability of 1D transport. Therefore, electrons can transport at a similar rate regardless of the number of boundaries introduced by ZnO nanobelts, consequently ALD1.0 and ALD2.0 cells have similar *J*_sc_ and *η*. As expected, these characteristics should also be indicated in the IPCE spectra (Fig. 10). As compared to the cells without ALD coating, ALD cells have higher IPCE values. The IPCE value of ALD0.5 cell is the highest, and the ALD1.0 and ALD2.0 cells have similar values, which are consistent with the trend of *η*.

## Conclusions

In summary, a new type of photoanode architecture has been designed by adopting a composite film as the working electrode to fabricate DSSCs, in which ultralong ZnO nanobelts are introduced into TiO_2_ nanoparticles followed by coating a thin layer of TiO_2_ film using ALD technique. Reflectance and absorption spectra, BET, IPCE, and EIS studies indicate that an optimum amount of ZnO nanobelts in the composite of ZnO nanobelts/TiO_2_ nanoparticles can enhance the conversion efficiency by 61.4% compared to that of conventional TiO_2_ nanoparticle films. Once coating a proper thickness (4 nm) of TiO_2_ film with ALD to improve the interface contact within the whole system, the efficiency can be remarkably improved by 137%. We mainly attribute the excellent performance to the reduced interface recombination, fast electron transfer and increased carrier lifetime, although the synergistic effect of light scattering and surface area is also considered.

## Methods

### Synthesis of ultralong ZnO nanobelts

ZnO nanobelts were synthesized in the solution without using any substrates at 90 °C followed by annealing at 500 ^o^C for 2 hour. During the water bath reaction, a 250 ml beaker contained 150 ml solution composed of 25 mM zinc nitrate hexahydrate (Zn(NO_3_)_2_•6H_2_O, Aldrich, 98%), 25 mM hexamethylenetetramine (HMTA, Aldrich, 99%), 10 mM ammonium fluoride (NH_4_F, Aldrich, ≥99.99%) and 150 ml deionized water. After 2 hours of reaction, the solution was filled with white flocculent ZnO nanobelts. Upon completion of the reaction, ZnO nanobelts were separated by Buchner funnel, rinsed with deionized water, and then dried at 80 °C overnight.

### Fabrication of dye-sensitized solar cells

First, ZnO nanobelts were blended with P25 TiO_2_ nanoparticles with different weight ratios (from 0–20%). The ZnO nanobelt/TiO_2_ nanoparticle composites were coated onto the clear FTO glasses and used as films of photoanode by a doctor-blade method. The films were sintered at 500 °C for 2 hour to remove the organic solvent. Then the films were sensitized by soaking in an ethanol solution containing 0.5 mM N719 dye (Solaronix SA, Switzerland) for 10 hour at room temperature. A counter electrode was prepared by thermal decomposition of H_2_PtCl_6_ ethanol solution at 400 ^o^C for 20 min on a cleaned FTO substrate with a pre-drilled hole. To assemble the cell, a 60 μm thick Surlyn film was sandwiched between the dye-sensitized photoanode and counter electrode by hot pressing, and the electrolyte with 0.6 M 1-butyl-3- methylimidazolium iodide (BMII, Aldrich, 99%), 0.02 M I_2_ (Aldrich, 99%), 0.01 M tert-butylpyridine (Aldrich, 96%) and 0.1 M guanidinium thiocyanate (Aldrich, ≥97%) in acetonitrile/valeronitrile (85/15 in volume) was injected through the holes. The active area of the resulting cells exposed to light was approximately 0.28 cm^2^. To fabricate ALD-modified photoanodes, ZnO nanobelt/TiO_2_ nanoparticle composites were coated by a thin layer of TiO_2_ film using atomic layer deposition (ALD, Ensure NanoTech, Beijing, China), in which tetra(dimethylamino)titanium (TDMAT, Jiangsu Nata Opto-electronic Materials Co., Ltd., China) and H_2_O were used as precursors to react at 80 °C. The thickness of TiO_2_ layer was controlled precisely by adjusting the cycle number of precursors. Finally, the ALD coated films were sintered at 500 °C for 2 hour.

### Characterization and measurement

The morphology and microstructure of samples were characterized using field-emission scanning electron microscope (FESEM, Hitachi, SU8010) and high-resolution transmission electron microscope (HRTEM, FEI Tecnai G2 F20 S-TWIN TMP). The phase of products was checked by the X-ray diffractometer (XRD, D/MAX-III-B-40KV, Cu Kα radiation; *λ* = 0.15418 nm). The absorbance and diffuse reflectance were detected by UV-vis spectrophotometer (Shimadzu UV-3600). Dye loading amount of the photoanode was determined by measuring the light absorption of the desorbed dye from the photoanode in 0.1 M NaOH aqueous solution. Brunauer–Emmett–Teller (BET) specific surface areas were determined from nitrogen sorption isotherms that were performed on a BEL-SORPmini system (BEL Japan). Photocurrent-voltage (*J*–*V*) characteristics were measured using a Keithley 2400 source meter under simulated AM 1.5G illumination (100 mW/cm^2^) provided by a solar light simulator (Newport, 94043A). Incident photon-to-current conversion efficiency (IPCE) was measured as a function of wavelength under short circuit conditions (Newport, IQE-200). The electrochemical impedance spectroscopy (EIS) were measured with an electrochemical workstation (Autolab, PGSTAT 302N) in dark at open circuit with the alternative signal amplitude 5 mV and frequency range from 0.1 Hz to 10^5^ Hz. Measurements were performed at an applied open-circuit voltage in dark.

## Additional Information

**How to cite this article**: Lu, H. *et al.* Interface Engineering through Atomic Layer Deposition towards Highly Improved Performance of Dye-Sensitized Solar Cells. *Sci. Rep.* **5**, 12765; doi: 10.1038/srep12765 (2015).

## Supplementary Material

Supporting Information

## Figures and Tables

**Table 1:** Photovoltaic parameters of DSSCs based on pristine TiO_2_ nanoparticles and ZnO0.5, ZnO1.0, and ZnO2.0 composites photoanodes.

Sample	*V*_oc_ [V]	*J*_sc_ [mA/cm^2^]	*FF*[%]	*η*[%]
TiO_2_	0.70	7.74	53.6	2.90
ZnO0.5	0.74	9.67	65.8	4.68
ZnO1.0	0.75	7.57	66.7	3.79
ZnO2.0	0.79	5.39	68.9	2.95

**Table 2:** Electrochemical parameters and dye loading of DSSCs based on pristine TiO_2_ nanoparticles and ZnO0.5, ZnO1.0, and ZnO2.0 composites photoanodes.

Sample	*R*_s_ [Ω]	*R*_Pt_ [Ω]	*R*_ct_ [Ω]	*τ*_e_ [ms]	*Dye loading*[nmol/cm^2^]
TiO_2_	10.56	10.96	18.26	5.2	80.5
ZnO0.5	10.79	8.182	64.22	1.6	81.7
ZnO1.0	10.86	10.86	92.11	0.41	101.5
ZnO2.0	11.21	12.27	129.0	0.26	165.9

Measurements were performed at an applied open-circuit voltage in dark.

**Table 3:** Photovoltaic parameters of DSSCs based on ALD TiO_2_ nanoparticles and ALD0.5, ALD1.0, and ALD2.0 composites photoanodes.

Sample	*V*_oc_ [V]	*J*_sc_ [mA/cm^2^]	*FF*[%]	*η*[%]
ALD TiO_2_	0.73	11.74	66.2	5.72
ALD0.5	0.75	13.83	67.0	6.88
ALD1.0	0.76	11.94	68.3	6.22
ALD2.0	0.79	11.43	67.2	6.10

**Table 4:** Electrochemical parameters and dye loading of DSSCs based on ALD TiO_2_ nanoparticles and ALDO0.5, ALD1.0, and ALD2.0 composites photoanodes.

Sample	*R*_s_ [Ω]	*R*_Pt_ [Ω]	*R*_ct_ [Ω]	*τ*_e_ [ms]	*Dye loading*[nmol/cm^2^]
ALD TiO_2_	11.13	12.88	56.32	26.5	80.6
ALD0.5	12.09	8.256	87.44	29.9	96.6
ALD1.0	11.37	9.461	92.26	17.9	113.9
ALD2.0	10.93	14.36	186.17	12.6	147.3

## References

[b1] Zhang Q., Dandeneau C. S., Zhou X. & Cao G. Z. ZnO nanostructures for dye-sensitized solar cells. Adv. Mater. 21, 4087–4108 (2009).

[b2] Yu M., Long Y. Z., Sun B. & Fan Z. Y. Recent advances in solar cells based on one-dimensional nanostructure arrays. Nanoscale 4, 2783–2796 (2012). 22473573 10.1039/c2nr30437f

[b3] Hagfeldt A., Boschloo G., Sun L., Kloo L. & Pettersson H. Dye-sensitized solar cells. Chem. Rev. 110, 6595–6663 (2010). 20831177 10.1021/cr900356p

[b4] Mathew S. *et al.* Dye-sensitized solar cells with 13% efficiency achieved through the molecular engineering of porphyrin sensitizers. Nat. Chem. 6, 242–247 (2014). 24557140 10.1038/nchem.1861

[b5] Kopidakis N., Benkstein K. D., Lagemaat J. & Frank A. J. Transport-limited recombination of photocarriers in dye-sensitized nanocrystalline TiO_2_ solar cells. J. Phys. Chem. B 107, 11307–11315 (2003).

[b6] Xu F. & Sun L. Solution-derived ZnO nanostructures for photoanodes of dye-sensitized solar cells. Energy Environ. Sci. 4, 818–841 (2011).

[b7] Zhuge F. *et al.* Toward hierarchical TiO_2_ nanotube arrays for efficient dye-sensitized solar cells. Adv. Mater. 23, 1330–1334 (2011). 21400591 10.1002/adma.201003902

[b8] Ahn S. H., Kim D. J., Chi W. S. & Kim J. H. Hydrophobic sponge structure-based triboelectric nanogenerator. Adv. Mater. 26, 5037–5042 (2014). 24848446 10.1002/adma.201401184

[b9] Labouchere P. *et al.* Tetreault. Passivation of ZnO nanowire guests and 3D inverse opal host photoanodes for dye-sensitized solar cells. Adv. Energy Mater. 4, 1400217 (2014).

[b10] Ozgur U. *et al.* A comprehensive review of ZnO materials and devices J. Appl. Phys. 98, 041301 (2005).

[b11] Tang H., Prasad K., Sanjines R., Schmid P. E. & Levy F. Electrical and optical properties of TiO_2_ anatase thin films. J. Appl. Phys. 75, 2042–2047 (1994).

[b12] Wang Z. L. Nanostructures of zinc oxide. Mater. Today 7, 26–33 (2004).

[b13] Chou T. P., Zhang Q. F. & Cao G. Z. Effects of dye loading conditions on the energy conversion efficiency of ZnO and TiO_2_ dye-sensitized solar cells. J. Phys. Chem. C 111, 18804–18811 (2007).

[b14] Westermark K. *et al.* PES studies of Ru (dcbpyH_2_)_2_(NCS)_2_ adsorption on nanostructured ZnO for solar cell applications. J. Phys. Chem. B 106, 10102–10107 (2002).

[b15] Bai Y. *et al.* *In situ* growth of a ZnO nanowire network within a TiO_2_ nanoparticle film for enhanced dye-sensitized solar cell performance. Adv. Mater. 24, 5850–5856 (2012). 22930471 10.1002/adma.201201992

[b16] Pang S. *et al.* Research on the effect of different sizes of ZnO nanorods on the efficiency of TiO_2_-based dye-sensitized solar cells. J. Phys. Chem. C 111, 18417–18422 (2007).

[b17] Sheng J. *et al.* Characteristics of dye-sensitized solar cells based on the TiO_2_ nanotube/nanoparticle composite electrodes. J. Mater. Chem. 21, 5457–5463 (2011).

[b18] Yeh M. *et al.* Preparing core–shell structure of ZnO@TiO_2_ nanowires through a simple dipping-rinse-hydrolyzation process as the photoanode for dye-sensitized solar cells. Nano Energy 2, 609–621 (2013).

[b19] Wu W. *et al.* Hierarchical oriented anatase TiO_2_ nanostructure arrays on flexible substrate for efficient dye-sensitized solar cells. Sci. Rep. 3, 1892 (2013). 23715529 10.1038/srep01892 PMC3665963

[b20] Ahn J. Y. *et al.* Designed synthesis and stacking architecture of solid and mesoporous TiO_2_ nanoparticles for enhancing the light-harvesting efficiency of dye-sensitized solar cells. ACS Appl. Mater. Inter. 6, 903–909 (2014). 10.1021/am4041866 24377279

[b21] Zhang S., Yang X., Numata Y. & Han L. Highly efficient dye-sensitized solar cells: progress and future challenges. Energy Environ. Sci. 6, 1443–1464 (2013).

[b22] Ohsaki Y. *et al.* Dye-sensitized TiO_2_ nanotube solar cells: fabrication and electronic characterization. Phys. Chem. Chem. Phys. 7, 4157–4163 (2005). 16474882 10.1039/b511016e

[b23] Bisquert J. Theory of the impedance of electron diffusion and recombination in a thin layer. J. Phys. Chem. B 106, 325–333 (2002).

[b24] Ahn S. H., Chi W. S., Kim D. J., Heo S. Y. & Kim J. H. Honeycomb-like organized TiO_2_ photoanodes with dual pores for solid-state dye-sensitized solar cells. Adv. Funct. Mater. 23, 3901–3908 (2013).

[b25] Chen L. *et al.* Enhanced photovoltaic performance of a dye-sensitized solar cell using grapheme-TiO_2_ photoanode prepared by a novel *in situ* simultaneous reduction-hydrolysis technique. Nanoscale 5, 3481–3485 (2013). 23483083 10.1039/c3nr34059g

[b26] Wang W. *et al.* FeSe_2_ films with controllable morphologies as efficient counter electrodes for dye-sensitized solar cells. Chem. Commun. 50, 2618–2620 (2014). 10.1039/c3cc49175g 24468707

[b27] Ye M. *et al.* Hierarchically structured nanotubes for highly efficient dye-sensitized solar cells. Adv. Mater. 25, 3039–3044 (2013). 23450829 10.1002/adma.201205274

[b28] Chen C., Li Y., Sun X., Xie F. & Wei M. Efficiency enhanced dye-sensitized Zn_2_SnO_4_ solar cells using a facile chemical-bath deposition method. New J. Chem. 38, 4465–4470 (2014).

[b29] Fang W. Q. *et al.* An efficient DSSC based on ZnO nanowire photo-anodes and a new D-π-A organic dye. Energy Environ. Sci. 4, 2903–2908 (2011).

[b30] Wu J., Liao W. & Yoshimura M. Soft processing of hierarchical oxide nanostructures for dye-sensitized solar cell applications. Nano Energy 2, 1354–1372 (2013).

[b31] George S. M. Atomic layer deposition: an overview. Chem. Rev. 110, 111–131 (2010). 19947596 10.1021/cr900056b

[b32] Meng X., Yang X. & Sun X. Emerging applications of atomic layer deposition for lithium-ion battery studies. Adv. Mater. 24, 3589–3615 (2012). 22700328 10.1002/adma.201200397

[b33] Knez M., Nielsch K. & Niinistö L. Synthesis and surface engineering of complex nanostructures by atomic layer deposition. Adv. Mater. 19, 3425–3438 (2007).

[b34] Kim D. H., Woodroof M., Lee K. & Parsons G. N. Atomic layer deposition of high performance ultrathin TiO_2_ blocking layers for dye-sensitized solar cells. ChemSusChem 6, 1014–1020 (2013). 23720440 10.1002/cssc.201300067

[b35] Dai H. *et al.* Porous ZnO nanosheet arrays constructed on weaved metal wire for flexible dye-sensitized solar cells. Nanoscale 5, 5102–5108 (2013). 23644717 10.1039/c3nr34265d

[b36] Bakke J. R., Pickrahn K. L., Brennan T. P. & Bent S. F. Nanoengineering and interfacial engineering of photovoltaics by atomic layer deposition. Nanoscale 3, 3482–3508 (2011). 21799978 10.1039/c1nr10349k

[b37] Lee S. W. *et al.* Improved Cu_2_O-based solar cells using atomic layer deposition to control the Cu oxidation state at the p-n junction. Adv. Energy Mater. 4, 1301916 (2014).

