# Supplementary material for: Interface Engineering through Atomic Layer Deposition towards Highly Improved Performance of Dye-Sensitized Solar Cells
Source: Sci Rep. 2015 Aug 4;5:12765. doi: 10.1038/srep12765 (PMC4523832; doi:10.1038/srep12765)
Supplement: Supporting Information [file srep12765-s1.pdf]

Supporting Information

Interface Engineering through Atomic Layer Deposition towards Highly Improved Performance of Dye-Sensitized Solar Cells

Hao Lu,[a] Wei Tian,[a] Jun Guo,[b] and Liang Li*[a]

[a]*College of Physics, Optoelectronics and Energy, Jiangsu Key Laboratory of Thin Films, Soochow University, China*

[b]*Analysis and Testing Center of Soochow University, Soochow University, China*

Email: lli@suda.edu.cn, liang.li0216@gmail.com

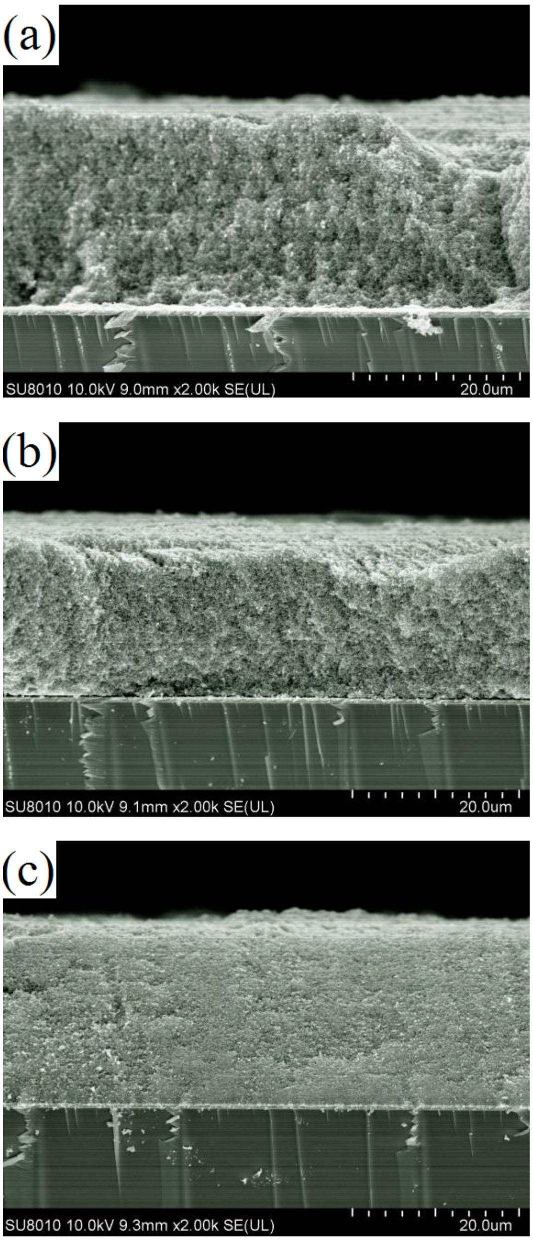

Figure S1. Cross-sectional SEM images for TiO_2 (a), ALD1.0 (b) and ALD2.0 (c) samples.

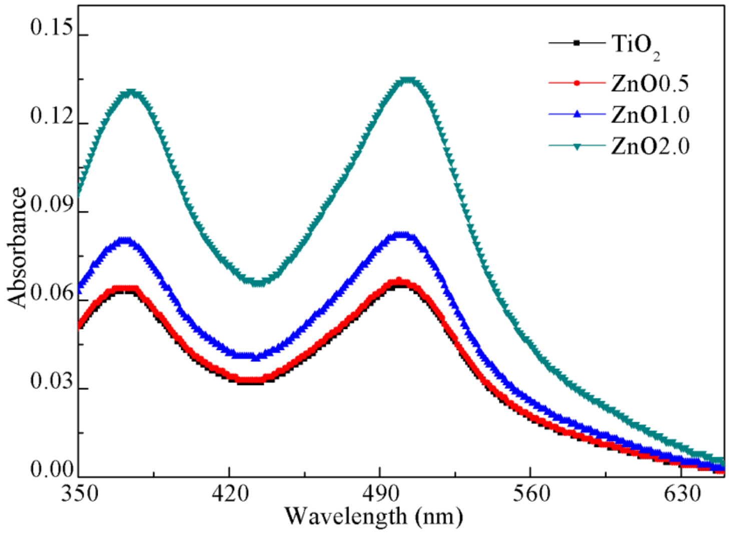

Figure S2. UV-Vis absorbance spectra of solutions containing dyes desorbed from the sensitized photoanodes composed of pristine TiO_2 nanoparticles and ZnO0.5, ZnO1.0, and ZnO2.0 composites.

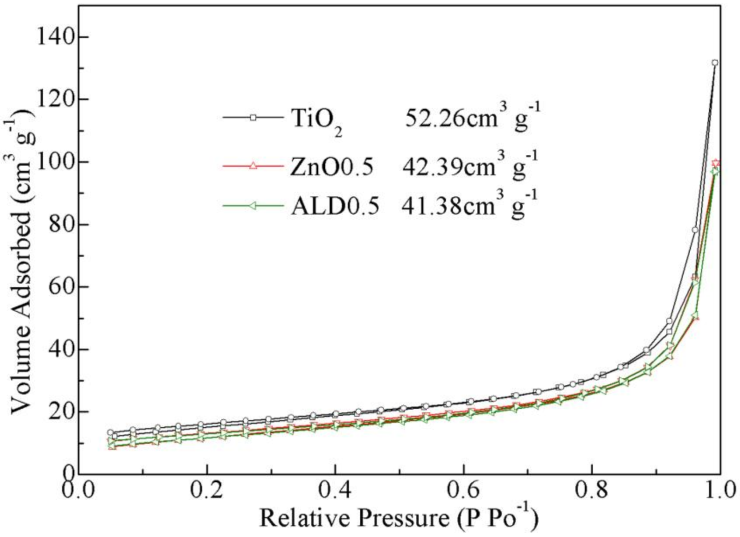

Figure S3. Nitrogen adsorption–desorption isotherms of pristine TiO_2 nanoparticles and ZnO0.5, ZnO1.0, and ZnO2.0 films.

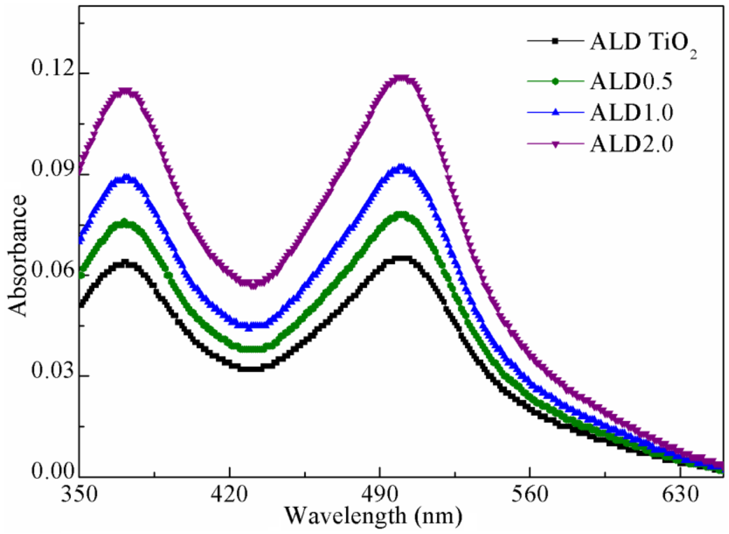

Figure S4. UV-Vis absorbance spectra of solutions containing dyes desorbed from the sensitized photoanodes composed of ALD TiO_2 nanoparticles and ALDO0.5, ALD1.0, and ALD2.0 composites.
